# The use of a robotic gait simulator for the development of an alignment tool for lower limb prostheses

**DOI:** 10.1186/1757-1146-7-S1-A15

**Published:** 2014-04-08

**Authors:** Eveline De Raeve, Tom Saey, Luiza Muraru, Louis Peeraer

**Affiliations:** 1 MOBILAB, University College Thomas More, Geel, Belgium; 2 BMe, Department of Mechanical Engineering, KU Leuven, Leuven, Belgium; 3 Faculty of Kinesiology and Rehabilitation Sciences, KU Leuven, Leuven, Belgium

## Aim

An innovative tool to optimise the configuration and alignment of lower leg prostheses based on individual comfort needs of the patient will be developed in this project.

## Background

This tool meets the demand of prosthetists to optimise and objectify the dynamic alignment of transtibial prostheses. Nowadays, the prosthetists mainly rely on their own expertise and experience.

Current methods to align the prosthesis start with a static alignment of transtibial prosthesis. This method does not take the individual patient comfort into account. Afterwards, adjustments to the alignment are done by trial and error. This is a time-consuming and exhausting activity for both prosthetist and patient.

## Methods

**Figure 1:**
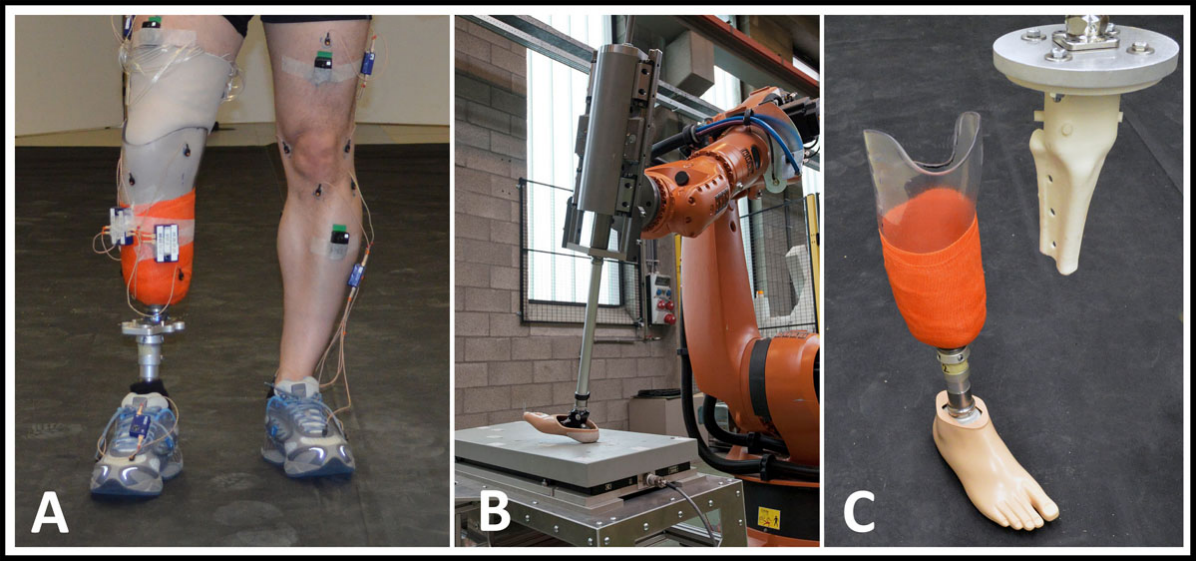
A. gait analyses of an amputee, B. robotic gait simulator, C. artificial stump

10 amputees were asked to walk with 20 different alignments and a neutral alignment (Figure 1A). The effect on comfort was questioned and measured. Therefore ground reaction force, 3D movement of the foot, the shank, upper leg, pelvic and torso and muscle activity were recorded with a force plate (AMTI), a motion tracking system (Codamotion) and EMG-sensors (Delsys). Simultaneously, contact pressure between stump and socket was recorded on 32 reference points with pressure sensors (mFLEX). All data was recorded synchronously at 200Hz.

Subsequently, the 3D movement of the shank was recalculated to Euler angles to be used as kinematic input for an industrial robot with 6 degrees of freedom (KUKA) (Figure 1B). An energy consumption system with spring enables us to simulate kinematic and kinetics of prosthetic gait. To objectively measure the pressure, an artificial stump was developed to connect the lower limb prosthesis to the robot (Figure 1C). This artificial stump, which consists of SLS-3D printed bones and soft tissue (silicon) allows us to objectively measure the pressure between stump and socket.

For different alignments, forces (AMTI), motion trajectories and contact pressure between stump and socket (mFLEX) were registered simultaneously during the unroll of the prosthetic foot. These measurements are translated into comfort parameters during post-processing.

## Results and discussion

Using our robotic gait simulator, the effect of prostheses alignment on patient comfort is mapped. The biggest advantage of this approach is the possibility to test numerous and extreme alignments, without the need for test subjects. After validation with gait analyses, this data is translated in an algorithm which will be converted into a useful method as a tool for the prosthetist.

The outcome of this project will lead to an improved efficiency and effectiveness of the alignment process, and contribute to the quality of life of the amputee.

## Trial registration

Clinical Trial Center - CTC 55509

